# Pixelating Responsive Structural Color via a Bioinspired Morphable Concavity Array (MoCA) Composed of 2D Photonic Crystal Elastomer Actuators

**DOI:** 10.1002/advs.202300347

**Published:** 2023-02-15

**Authors:** Yi Pan, Chang Li, Xiaoyu Hou, Zhenyu Yang, Mingzhu Li, Ho Cheung Shum

**Affiliations:** ^1^ Department of Mechanical Engineering The University of Hong Kong Pokfulam Road Hong Kong 999077 P. R. China; ^2^ Key Laboratory of Green Printing Institute of Chemistry Chinese Academy of Sciences Beijing 100190 P. R. China; ^3^ Advanced Biomedical Instrumentation Centre Hong Kong Science Park New Territories, Shatin Hong Kong 999077 P. R. China

**Keywords:** 2D photonic crystal, elastomer actuator, morphable concavity, pixelating, structural color

## Abstract

Stimuli‐responsive structural coloration allows the color change of soft substrates in response to environmental stimuli such as heat, humidity, and solvents. Such color‐changing systems enable smart soft devices, such as the camouflageable skin of soft robots or chromatic sensors in wearable devices. However, individually and independently programmable stimuli‐responsive color pixels remain significant challenges among the existing color‐changing soft materials and devices, which are crucial for dynamic display. Inspired by the dual‐color concavities on butterfly wings, a morphable concavity array to pixelate the structural color of two‐dimensional photonic crystal elastomer and achieve individually and independently addressable stimuli‐responsive color pixels is designed. The morphable concavity can convert its surface between concave and flat upon changes in the solvent and temperature, accompanied by angle‐dependent color‐shifting. Through multichannel microfluidics, the color of each concavity can be controllably switched. Based on the system, the dynamic display by forming reversibly editable letters and patterns for anti‐counterfeiting and encryption are demonstrated. It is believed that the strategy of pixelating optical properties through locally altering surface topography can inspire the design of new transformable optical devices, such as artificial compound eyes or crystalline lenses for biomimetic and robotic applications.

## Graphical abstract

Inspired by the dual‐color concavities on butterfly wings, a morphable concavity array is designed to pixelate the structural color of a two‐dimensional photonic crystal elastomer. The concavity can convert its surface between concave and flat upon changes in the solvent and temperature, associated with angle‐dependent color‐shifting, which facilitates the dynamic display by forming reversibly editable letters and patterns.

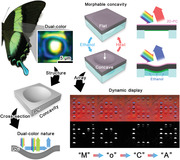

## 1 Introduction

Natural creatures, such as cephalopods and chameleons, can adapt their color or pattern to signal their appearance amongst others or blend in with their surroundings.^[^ ^1^ ^]^ This dynamic color‐changing or patterning feature in their soft and elastic skins has spurred the invention of artificial color‐changing soft matter systems for flexible displays or camouflaging.^[^ ^1^, ^2^ ^]^ Color switch of these systems is triggered by changes in environmental stimuli, such as heat, moisture, and solvents.^[^ ^2^, ^3^ ^]^ Two common types of stimuli‐responsive colors are induced by changes in the chemical properties of pigments or dyes and the periodic nanostructures of structural color systems.^[^ ^2c^ ^]^ For constructing a soft color‐changing system, structural color offers a broader range of hues and higher durability against photochemical degradation.^[^ ^1^, ^2^, ^3^ ^]^ Typical responsive structurally colored materials comprise photonic crystal (PC) structures, such as periodically stacked nanoparticles, and a stimuli‐responsive matrix, such as hydrogel or elastomer with solvent‐ or temperature‐triggered volume change.^[^ ^1^, ^2^ ^]^ Along with the volume change of the matrix, the structural color also changes because of the varying lattice constant of the PC structure.^[^ ^1^, ^2^ ^]^ Based on similar methods, numerous soft substrates endowed with responsive structural colors or preset patterns are utilized for colorimetric detection and anti‐counterfeiting.^[^ ^1^, ^2^, ^4^ ^]^ These environment‐responsive structural colorations also open up more possibilities, especially in all‐soft‐matter color‐changing systems, representing a complementary approach to the conventional electrochromic methods. To create arbitrarily switchable patterns that resemble their natural counterparts and match the functionality of their electronic counterparts requires more than just overall color changes with unaltered patterns. Dividing the responsive structural color substrate into smaller units that can be regulated individually allows editable patterns. In this way, a more sophisticated and programmable color‐changing system can be realized and applied in dynamic displays.

Pointillism was first proposed by visual artists as a method of composing patterns through dots and is now widely adopted by electronic and digital display technologies.^[^ ^5^ ^]^ In displaying a digital image, the image is cut into arrays of uniform dots, and the color of each dot, known as a pixel, is independently controlled. Likewise, converting the stimuli‐responsive structural color to pixels allows the creation of editable patterns.^[^ ^2^, ^6^ ^]^ Nonetheless, pixelating a complete piece of stimuli‐responsive PC material remains challenging. This challenge mainly stems from partitioning the responsive material into pixels and independently controlling those pixels by the stimuli. Typically, the partitioning methods can be categorized into a bottom‐up strategy, i.e., assembling small pieces of material into a system, and a top‐down strategy, i.e., dividing a complete material into small regions.^[^ ^2f^ ^]^ Compared to the bottom‐up approach, the top‐down approach avoids the tedious assembly process and piecemeal components, retaining the completeness of the material and facilitating mass production.^[^ ^7^ ^]^ In collaboration with effective partitioning, independently controllable stimulation can be more readily carried out. In principle, modulating the optical signals via stimulus‐triggered response consists of three main approaches: varying the refractive indices, manipulating the photonic nanoarchitectures, and inclining the glancing angle.^[^ ^1^, ^2^, ^8^ ^]^ Pixelation based on the two former approaches requires precisely adjusting the PC properties of a certain local region within the complete material. However, for some environmental stimuli, such as liquid and heat, it is difficult not to blur the region edges due to the dynamic diffusion, such as penetration of fluids or heat transfer. Thus, the approach of inclining the glancing angle, which involves only the deformation of the material, is more general because it has fewer restrictions on the stimulus type. For example, by the kirigami strategy, planar films can transform into 3D architecture via the out‐of‐plane bending of the cut units in response to force, light, or magnetism.^[^ ^7^, ^9^ ^]^ The out‐of‐plane kirigami has provided a successful template for pixelating the color of the PC. Nevertheless, this unidirectionally deformed structure limits the orientation where the color change can be observed as well. Hence, alternative methods need to be developed to complement each other. In particular, a facile and effective morphing design of the responsive structural colors is still worth exploring for their pixelation.

**Figure 1:**
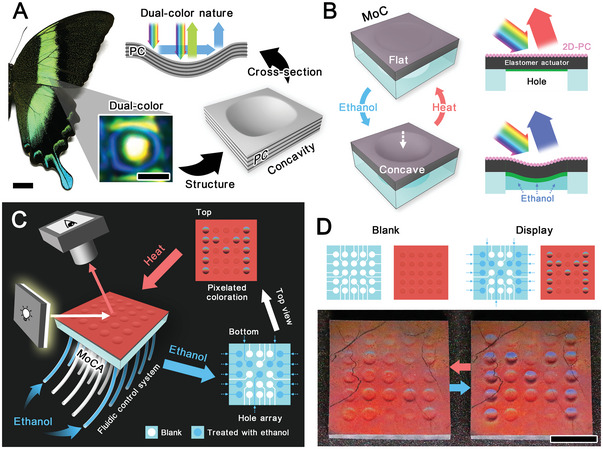
Bio‐inspired design of MoCA. A) The dual‐color nature of the concavity in the scales of the *Papilio palinurus* butterfly wing and its dual‐color mechanism. Scale bar: 1 cm. Inset: a real‐color image of one concavity on the wing scale. Scale bar: 5 µm. B) Schematic illustration depicting the transformation of the MoC's surface topography and its color‐changing mechanism. C) Schematic illustration of the set‐up for the observation of color change of MoCA. D) Schematic and photography of the pattern formation on MoCA.

In nature, the *Papilio palinurus* butterfly exhibits dual color on its wing scales due to the micro‐concavities with PC structure (**Figure** **1**A and Figure S1, Supporting Information).^[^ ^10^ ^]^ Specifically, when a beam of light is incident on the concavity, the crystal orientation disparity between the center and edge leads to different angles of incidence and, thus, distinct reflection wavelengths.^[^ ^10^, ^11^ ^]^ Therefore, though the PC structures in the butterfly's wings are consistent, the variations in surface topography can also derive distinct colors. Inspired by this PC concavity's angle‐dependent color, we design a morphable concavity (MoC) composed of two‐dimensional photonic crystal (2D‐PC) and elastomer actuators (EA) to pixelate the solvent‐responsive structural color (Figure 1B). The MoC can locally alter the color of the photonic crystal elastomer actuator (PC‐EA) by forming cylindrosymmetric concave surfaces in response to solvent. The actuator layer at the bottom of MoC can deform owing to internal stress triggered by solvent, while the 2D‐PC on the upper surface forms a concavity. Since the color of 2D‐PC is angle‐dependent, the color of the concave area varies with its surface topography change. A single MoC can be employed as a responsive color‐changing pixel, and then a MoC array (MoCA) can form a display (Figure 1C). By manipulating each MoC in MoCA via microfluidics, a particular pattern can be composed (Figure 1D). We further demonstrate its dynamic display and use it for distinctive anti‐counterfeiting and encryption, proving the immense potential of integrating morphological flexibility with the optical performance. This concept of pixelating optical properties through altering surface topography can also benefit the design of transformable optical components in biomimetics and soft robotics, such as artificial compound eyes and crystalline lenses.

## 2 Results and Discussions

### 2.1 Preparation and Characterization of the MoCA

**Figure 2:**
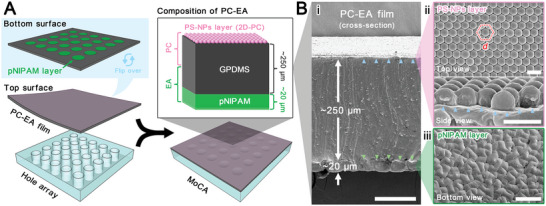
Structure of MoCA. A) Structural composition of MoCA. B) SEM images of PC‐EA film at the concavity area. (i) Cross‐section with interfaces between PS‐NPs and GPDMS (marked with blue arrows) and between GPDMS and pNIPAM (marked with green arrows). Scale bar: 100 µm. (ii) Top and side views of the PS‐NPs layer. Scale bars: 1 µm. (iii) Top views of the pNIPAM layer. Scale bars: 100 µm.

The MoCA is fabricated by combining PC‐EA film and an elastomeric partition with a hole array (**Figure** **2**A). First, a PC elastomer film is prepared by assembling a monolayer of polystyrene nanoparticles (PS‐NPs) onto a graphene nanoplates and benzophenone (BP) codoped polydimethylsiloxane (PDMS) elastomer film (GPDMS) surface (Figure S2A, Supporting Information). The graphene nanoplates are used to provide a black background, enhancing the contrast of the color. The BP functions as an oxygen inhibitor and a UV‐triggered initiator for the subsequent radical polymerization of hydrogels. Then a hole array is bonded to the back of the PC elastomer film, followed by modifying the solvent‐responsive hydrogel, poly(N‐isopropylacrylamide) (pNIPAM), into the hole. During the pNIPAM modification, N‐isopropylacrylamide (NIPAM) monomers with cross‐linkers are first applied on the GPDMS surface. By subsequent UV excitation, triplet state BP in the GPDMS is produced and abstracts a hydrogen atom from the methyl group of PDMS to yield radicals. These radicals then initiate the NIPAM graft polymerization on the near surface of GPDMS, forming a thin pNIPAM substratum in the wells (Figure S2B, Supporting Information). Finally, a MoCA device composed of 2D‐PC, GPDMS, pNIPAM, and a hole array from top to bottom is obtained. The upper layer of 2D‐PC generates structural colors, while the lower partitioned solvent‐responsive layer serves as a transformable hydrogel‐elastomer bilayer actuator. Such a hybrid structure of the actuator can preserve the inherent properties of the soft hydrogel while being mechanically reinforced by the tough elastomer. In addition, the water‐rich hydrogel will collapse structurally due to water evaporation from prolonged exposure to air, while the water‐free elastomer can then support the actuator structure as a skeleton.^[^ ^12^ ^]^ When illuminated with natural light, the upper surface of PC‐EA is black due to the GPDMS substrate (Figure S3, Supporting Information). However, under the omnidirectional incident light at a view angle of 20°, the PC‐EA surface displays brilliant red color due to the 2D‐PC with nanoscaled structural periodicity (Figure 1D). The bottom of the device, which is an elastomeric partition with a hole array to first support the PC‐EA layer to prevent excessive deformation, functions as a solvent reservoir and separates the adjacent responsive areas of MoC. From the scanning electron microscope (SEM) image, we can observe the cross‐section of the device, which has a total PC‐EA film thickness of ≈270 µm (Figure 2Bi). During the assembly of hydrophobic PS‐NPs on the water surface, the inconsistent spreading rate of PS‐NPs results in the nonhomogeneity of the local particle density (Figure S2Aii, Supporting Information). This nonhomogeneity also leads to local defects within the large‐area ordered assembly of PS‐NPs on GPDMS (Figure S4A, Supporting Information). In the ordered area, PS‐NPs are close‐packed hexagonal with a lattice constant *d* of 600 nm (Figure 2Bii). PS‐NPs are slightly embedded in the GPDMS (Figure 2Bii and Figure S4B, Supporting Information), preventing the PS‐NPs layer on the elastomer from getting delaminated easily in the absence of external forces. In the defective areas, PS‐NPs appear as multilayered or poorly packed stacks (Figure S4A, Supporting Information). These small defects would lead to discontinuities in the color rendering of individual microscopic regions in subsequent experiments. Nonetheless, they do not exhibit abrupt colors or invalidate the stimuli‐responsive coloration in our device, especially in macroscopic visual performance. At the bottom of the PC‐EA film, a wrinkled pNIPAM layer is combined with GPDMS by chemical bonding (Figure 2Bi and Figure 2Biii). The energy‐dispersive X‐ray (EDX) mapping on the cross‐section of the device reveals that the abundant silicon (Si) only exists at the middle layer, which comes from the siloxane backbone of PDMS (Figure S4B, Supporting Information). Hence, we identify the interfaces between the PS‐NPs layer and GPDMS as well as GPDMS and pNIPAM (Figure 2Bi and Figure S4B, Supporting Information). These analysis results prove that PC‐EA is a trilayer structure consisting of a PS‐NPs layer, GPDMS layer (250 µm in thickness), and pNIPAM layer (20 µm in thickness) in a stack.

### 2.2 Responsive Morphing of the MoC

**Figure 3:**
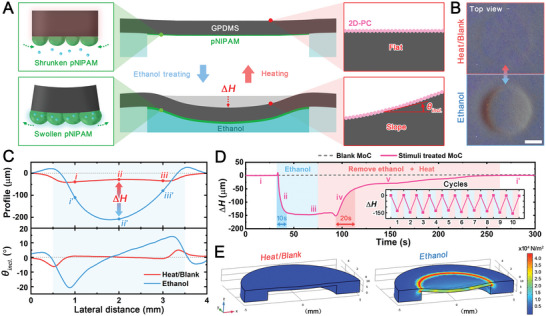
Stimuli‐responsive topographical change of the MoC. A) Schematic illustration of the stimuli‐responsiveness of MoC (cross‐section). *θ*_*incl*._ is defined as the angular magnitude of the counterclockwise rotation of the right end of the initial plane under study in the observation field. B) Photographs of the erasure and appearance of the concavity (top view) triggered by stimuli. Scale bar: 1 mm. C) Profiles and the corresponding *θ*_*in* *cl*_ *_._* of the concave area (indicated by blue background) in its heating/blank state and ethanol‐treated state. *i*, *ii*, and *iii* (or *i’*, *ii’*, and *iii’*) are the three studied points equidistantly distributed on the profiles. D) Dynamic change of the *∆H* without and with stimuli treatment (light blue and light red backgrounds denote the ethanol treating period and the combined period of ethanol removal and heat treatment at 60 °C, respectively). Inset: Reproducibility test of *∆H* falling and rising under alternating stimuli. E) Finite element analysis of the strain distribution on the MoC (oblique top‐down view).

The responsive morphing of MoC is determined by the hydrogel‐elastomer bilayer actuator which is bonded by a pNIPAM layer actively responding to the stimuli and a GPDMS layer passively responding to the same stimuli. Specifically, the disparities in swelling rate between the pNIPAM and the GPDMS in response to the solvent enable the morphing response in the MoC (**Figure** **3**).^[^ ^12^ ^]^ When exposed to ethanol, pNIPAM can absorb the ethanol and swell more rapidly than GPDMS (Figure 3A, S5, and Table S1, Supporting Information).^[^ ^13^ ^]^ The expansion of pNIPAM is restricted by the GPDMS, so if the bonding between pNIPAM and GPDMS is sufficiently tight, the pNIPAM layer is subject to tension while the GPDMS layer is under compressive stress, leading to internal tensions.^[^ ^12^ ^]^ Consequently, the MoC structure is buckled downward to release the internal tensions. While releasing tensions, MoC is restricted by circular elastomeric partitions. As a result, the circumferential deformation is not obvious, but the deformation at the center is significant, creating a concave shape. The concave MoC can then revert to a flattened geometry as the ethanol is removed via microchannel and evaporates completely. This transformation of MoC can be preliminarily revealed by its shadows and reflections. From the top view, we cannot observe shadows or reflections cast by the surrounding light on the blank MoC. After ethanol's stimulation of the actuator layer, distinct shadows and reflections can be observed (Figure 3B and Movie S1, Supporting Information). This phenomenon suggests that the topography of the MoC surface switches between flat and concave, inducing light and shadow alterations. During the MoC's topography transition, the 2D‐PC on its local surface will become inclined at an angle (*θ_incl._*) (Figure 3A). The profile through the center of symmetry can be traced to quantify the concavity and *θ_incl._*, since the MoC structure is designed to be cylindrosymmetric. We compare the profiles of the upper surface in its heating/blank state and its ethanol‐treated state (Figure 3C). In the blank state, the MoC area sinks slightly to form a “dish shape” with a sloping circumference and a flat center due to the inherent humidity in the air. Its corresponding inclination angle on the profile ranges from −6° to 5°. After ethanol treatment, the height at the center drops significantly, while the circumferential edge is only slightly raised, leading to a “bowl shape”. The inclination angle is widened from −21° to 14°. We monitor the MoC center point height change (*∆H*) in real time upon adding ethanol and following ethanol removal by microchannel plus 60 °C heating (Figure 3D). After being triggered by ethanol, *∆H* drops ≈150 µm in 10 s. The subsequent ethanol removal and heating allow *∆H* to start rising in a gradient. In stage iv, the ethanol near the surface of the pNIPAM evaporates rapidly, causing it to shrink, so *∆H* climbs by 100 µm in 20 s. The small amount of ethanol remaining deep inside the pNIPAM takes ≈2 min to be completely expelled, so *∆H* rises slowly by 50 µm in stage v. Furthermore, the falling and rising of *∆H* in response to alternating stimuli is reproducible, demonstrating the robustness of the MoC's transformation (Figure 3D inset). With an increase in ethanol concentration, the *∆H* gradually increases, associating with the rising inclination angle range (Figure S6A, Supporting Information). While the ethanol concentration varies from 0 to 50%, *∆H* changes slightly. However, there is a significant drop when the ethanol concentration is increased from 50 to 80%, and *∆H* reaches its lowest point at 100% (Figure S6B, Supporting Information). This is because pNIPAM has a higher swelling degree for the higher concentration of ethanol solution, resulting in more pronounced deformation.^[^ ^13a,b,13f^ ^]^ The MoC is also sensitive to temperature changes. When the ambient temperature is higher, the concave MoC flattens faster because the high temperature accelerates the evaporation of ethanol from the pNIPAM layer (Figure S7, Supporting Information). The rate of ethanol removal from the pNIPAM layer essentially determines the rate of MoC flattening. Therefore, if the ambient temperature is constant, the response time of MoC flattening can also be shortened by other approaches, such as enhancing the ambient airflow circulation and introducing local photothermal radiation. The disparities in the swelling rate between the pNIPAM and the GPDMS cause a nonuniform stress distribution along with the film. As supported by a finite element analysis model, the circumference of the concave area has larger internal stress than the center area, thus leading to the formation of a “bowl shape” concavity and the hump at the circumference (Figure 3F, Figure S8, and Movie S2, Supporting Information). We adjust the geometric parameters of MoC in our simulations and find that a thicker pNIPAM layer and a thinner GPDMS layer would speed up the response rate of the MoC and increase *θ_incl._* under the same actuating conditions, while adjusting the MoC diameter shows a negligible effect, since the curvature of this pNIPAM‐GPDMS bilayer actuator is determined by the thicknesses of the pNIPAM as the active layer and the GPDMS as the passive layer rather than the size of the actuator (Figure S9, Supporting Information). In principle, when the active layer is designed to be thicker and the passive layer is designed to be thinner, the response time is required to reach a certain *θ_incl._* will be shorter. Thus, the layers of pNIPAM and GPDMS in our MoCA prototype are designed to be the thickest and thinnest, respectively, to achieve a pronounced responsive morphing based on the fabrication techniques and facilities accessible to us.

### 2.3 Angle‐Dependent Color Switching of the MoC

**Figure 4:**
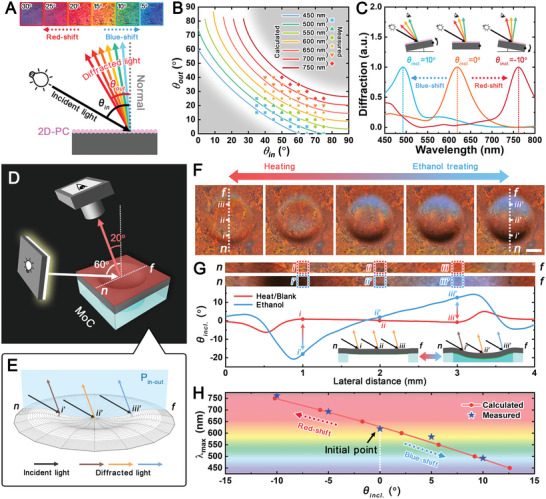
Color switching of the MoC. A) Schematic illustration shows the color shifts with various diffraction angles at a fixed incident angle within the plane containing the incident and diffracted lights for 2D‐PC. Inset: photographs of the 2D‐PC from different diffraction angles (*θ*_*out*_ = 5°–30°) at a fixed incident angle of 60°. B) The calculated (curves) and measured (dots) diffraction wavelengths (visible wavelength: *λ* = 450 nm–750 nm) in *θ*_*in*_ − *θ*_*out*_ diagram. The calculated curves are based on a nearest neighbor particle spacing of 600 nm. The measured dots are the wavelengths where the peaks of the spectra are located at the corresponding *θ*_*in*_ and *θ*_*out*_. The resulting peak wavelengths are categorized as the same icon at every 50 nm interval. (The gray background indicates the nonvisible area.) C) The 2D‐PC spectra at specific inclination angles. Insets: the schematic illustration shows the observed color changes with the inclination angle when the light source and observation position are constant. D) Schematic illustration of the set‐up for detecting color change of MoC. E) Schematic illustration shows the concavity subdivisions based on the finite element method. *n* and *f* represent the near‐end and far‐end of the concavity relative to the light source in the plane containing incidence and diffraction directions (P_in‐out_). *i*, *ii*, and *iii* are the three studied subdivided planes equidistantly distributed on concavity from *n* to *f* direction. The *θi*_*incl*_ *_._*, *θii*_*incl*_ *_._*, and *θiii*_*incl*_ *_._* denote the inclination angles of *i*, *ii*, and *iii*, respectively. F) Photographs of the color change of MoC (top view). Scale bar: 1 mm. G) Relationship between color and inclination angle at certain spots of MoC profile. The lateral observation points *i*, *ii*, and *iii* are 1, 2, and 3 mm, respectively. Inset: schematic illustration of the cross‐sections before and after the concavity appearance and light diffraction on the surface. H) The theoretically calculated and measured maximum wavelength of diffracted light in the visible region versus the inclination angle of the subdivided plane on MoC. Initial conditions: *θ*_*in*_ = 60° and *θ*_*out*_ = 20°.

The structural colors of MoC come from the PS‐NPs assembled into 2D‐PC which behaves as a 2D diffraction grating that diffracts light into various Bragg diffraction orders.^[^ ^7^, ^14^ ^]^ Thus, we can observe the diffracted light of this 2D‐PC on the same side of the incident light (**Figure** **4**A). The wavelength of its diffraction depends on both the incidence and diffraction angles, which means the color of 2D‐PC is angle‐dependent. The 2D‐PC diffraction follows the equation^[^ ^7^, ^14^ ^]^:

(1) $$d\frac{\sqrt{3}}{2}\;\left(\sin\theta_{out}+\sin\theta_{in}\right)=\;n\lambda$$

where *d* is the distance between two neighboring particles, *θ*_*in*_ and *θ*_*out*_ are the incidence and diffraction angles, *n* is the diffraction order, and *λ* denotes the wavelength of diffraction light. Based on the equation, the diffraction wavelength from 2D‐PC and the corresponding angular parameters can be closely related. Assuming a constant *θ*_*in*_, the color gradually redshifts or blueshifts as *θ*_*out*_ increases or decreases. For instance, when *θ*_*in*_ is fixed at 60°, the color of 2D‐PC converts from blue to red as *θ*_*out*_ increases from 5° to 30° (Figure 4A inset, S10A, and S10B, Supporting Information). In the chromaticity diagram, the PC elastomer shows that its color variation covers the whole visible range (Figure S10C, Supporting Information). An angle change of ≈15° can allow the color to switch between blue and a contrasting red. According to the 2D diffraction condition (Note S1, Supporting Information), the specific wavelength of the diffracted light in the visible region from 450 to 750 nm has been calculated and plotted in the *θ*_*in*_ − *θ*_*out*_ diagram (Figure 4B).^[^ ^14e^ ^]^ Moreover, we measure the spectra for a series of *θ*_*in*_ and *θ*_*out*_ separately and mark the wavelengths where their peaks are also located in the *θ*_*in*_ *− θ*_*out*_ diagram. The measured wavelengths of the diffracted light agree well with those calculated values, as shown in Figure 4B. Therefore, in the 2D‐PC coloration process, not only can we obtain the relationship between diffraction wavelength and angular parameters by experimental measurement, but also deduce a reliable diffraction wavelength with known incidence and diffraction angles. More importantly, we can apply the same theories and measurements to explain the diffracted light obtained by inclining the 2D‐PC plane when the direction of incidence and observation point remains constant (Figure 4C and Movie S3, Supporting Information). We take the farthest end of the 2D‐PC plane from the incident light as the reference point, as shown in Figure 4C inset. Then the angle by which the farthest end rotates counterclockwise in the plane containing the incident and diffracted directions (P_in‐out_) is defined as a positive *θ*_*incl*_ *_._*, and the angle after clockwise rotation is defined as a negative *θ*_*incl*_ *_._*. Based on the geometric relationship between *θ*_*in*_, *θ*_*out*_, and *θ*_*incl*._, the angles of incidence and diffraction after a certain inclination can be calculated as *θ’*_*in*_ = *θ*_*in*_ − *θ*_*incl*_ *_._* and *θ*’_*out*_ = *θ*_*out*_ − *θ*_*incl*._, respectively. Thus, we select the diffraction spectra with specific inclination angles from the measured spectral libraries (Figure 4C). At an initial *θ*_*in*_ of 60° and a *θ*_*out*_ of 20°, the peak will blueshift from 618 nm to 492 nm when *θ_incl._* increases from 0° to 10°. Inversely, the peak will redshift from 618 nm to 762 nm as *θ*_*incl*._ decreases from 0° to −10°. In all, the inclination of the 2D‐PC changes the angles of incidence and diffraction, which can in turn alter the observed color.

Based on the 2D‐PC's angle‐dependent optical property, we set the values of *θ*_*in*_ = 60° and *θ*_*out*_ = 20° to place the initial diffracted wavelength (≈600 nm) of MoC in the middle of the visible region (Figure 4D). According to the geometric characteristics of the concavity, the most dramatic shape and color changes will appear along a line *n*‐*f* that crosses through the symmetry point of the MoC surface and is in the P_in‐out_ plane (Figure 4E). Upon exposure to ethanol, the color of MoC changes initially from the edges near the *n* and *f* ends (Figure 4F and Movie S4, Supporting Information). The colored areas gradually converge toward the middle. Eventually, the concave area exhibits three distinct colors: blue near the *f* end, red in the middle, and black near the *n* end. It will turn back to the original red color as a whole after heating. The original color of MoC can be adjusted by the default angle parameters. For example, keeping *θ*_*in*_ constant at 60°, the original red, green, and blue MoC can be obtained by decreasing *θ*_*out*_ from 20° to 10° and then to 0° (Figure S11, Supporting Information). Although the apparent blue near the *f* end broadens as *θ*_*out*_ decreases, the strong contrast before and after the color switch is the key to color discrimination. Thus, we present MoC to switch between the two colors that are more distinct, i.e., red and blue, whose characteristic wavelengths are far apart. Consequently, color changes in both the MoC and MoCA can be readily detected, especially in their blue channel images (Figures S11 and S12, Supporting Information). Before explaining how the color of MoC changes in this way, we need to subdivide the morphing area into many small planes in a finite element manner (Figure 4E). Consequently, our quantitative analysis focuses on three representatives subdivided planes equidistantly distributed along line *n*‐*f*: *i*, *ii*, and *iii* (*i’*, *ii’*, and *iii’* refer to the corresponding positions after concavity is formed). The specific positions of *i*, *ii*, and *iii* are projected into the profile results in Figure 3C at 1, 2, and 3 mm of the MoC's lateral view, respectively. Then the equivalent points in the inclination angle curve of concavity profiles can be positioned and related to the color change area, as shown in Figure 4G. In a flat state, all three planes have *θ*_*incl*._ close to 0°. After the concavity is formed, their inclination angles are *θi’*_*incl*._ ≈ −18°, *θii’*_*incl*_ *_._* ≈ 2°, and *θiii’*_*incl*_ *_._* ≈ 13°. From the photographs, the color of *i* becomes black, the color change of *ii* is negligible, and the color of *iii* shows a significant blueshift. As long as the initial incidence and diffraction angles are known (*θ*_*in*_ = 60° and *θ*_*out*_ = 20°), we can calculate the observed diffraction wavelength distribution when the *θ*_*incl*_ *_._* varies from −20° to 20° (Note S2 and Figure S13, Supporting Information). A theoretical graph of maximum diffraction wavelength versus inclination angle can be obtained by extracting the intersection of the wavelength curves with the graph of function *θ’*_*out*_ = *θ’*_*in*_ − 40° in the *θ*_*in*_ − *θ*_*out*_ coordinates of Figure S13, Supporting Information. The initial wavelength is ≈625 nm, as shown in Figure 4H. When the variation of *θ’ii*_*incl*._ is less than 2.5°, the *λ*_*max*_ is between 600 and 650 nm, and the *ii* change thus is not apparent. Meanwhile, *θ’iii*_*incl*_ *_._* becomes 13°, spanning its *λ*_*max*_ to the blue region of 450 nm. The −18° of *θ’i*_*incl*_ *_._* pushes the *λ*_*max*_ step out into the invisible zone of near‐infrared; hence the black observed is the color of the graphene nanoplate in the elastomer film. The above calculations also corroborate our actual spectra measurement in the PC‐EA with different inclination angles. Since MoC's color depends on its topography, then the speed of color change corresponds to the rate of the MoC concaving and flattening upon stimulations. Combining the dynamic processes of changes in both color and *∆H* in MoC, we can observe that the response time of blue color appearing and disappearing in MoC matches the 10 s of stage ii and 20 s of stage iv in Figure 3D, respectively (Movie S4, Supporting Information). Moreover, we can infer that by adjusting the MoC structure parameters, such as thinning the GPDMS layer and thickening the pNIPAM layer, the color‐changing response time can be optimized accordingly. In addition, through the cyclic test, we also prove that the color‐switching of MoC is as repeatable as its transformation (Movie S5, Supporting Information). Thus, a single MoC can be taken as one color‐switching pixel and further assembled into a multipixel color‐changing system.

### 2.4 Programmable MoCA in Dynamic Display

**Figure 5:**
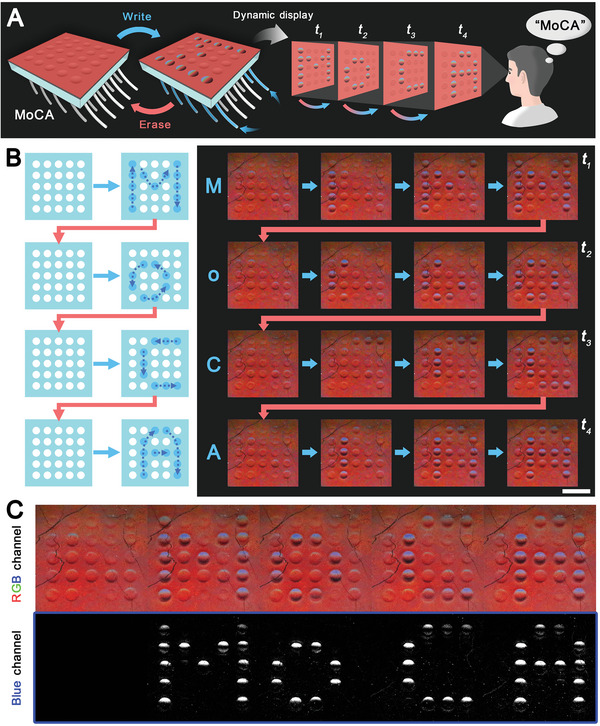
The dynamic display of MoCA. A) Schematic illustration shows the programmable color change of MoCA and its dynamic display application. B) Schematic illustrations and sequential images show the dynamic display of MoCA. Scale bar: 1 cm. C) MoCA's RGB images show the blank, letters “M”, “o”, “C”, and “A”, as well as their blue channel images after background correction.

Multiple controllable MoCs can be adopted as a series of color‐changing pixels and be arranged neatly into a color‐programmable pixel array termed MoCA. By triggering the color change of specific pixels on the array, the desired symbols or patterns can be formed dynamically. These colored patterns will eventually be acquired by the visual system and transformed into meaningful information for the viewers (**Figure** **5**A). We apply a multi‐channel microfluidic system to introduce the ethanol to certain holes beneath the MoCA device to deform the specific MoCs. The incorporation of microfluidics enables this color‐changing system to be more programmable in a dynamic display. After the treatment with ethanol in the order of alphabetic writing, the blue color in the specific pixels is gradually rendered following the writing order. It can eventually be distinguished from the original red color of the blank MoCA (Figure 5B and Movie S6, Supporting Information). After writing a letter, we can remove the ethanol via microfluidics and use heat to erase the blue pixels and then rewrite the next letter. In this way, different letters, such as “M”, “o”, “C”, and “A”, are dynamically displayed over time and combined into a meaningful word, “MoCA”, as shown in Figure 5C. Since the ethanol‐induced concavity is the reason for MoC's coloration, the letter or pattern will be stable as long as the presence of ethanol in specific MoCs can be maintained by microfluidic manipulation. Once the ethanol is removed to flatten the MoCs, the color will return to its default background color and thus the letter or pattern will be cleared. In this system, the appearing blue dots are the pixels that contain useful information. Hence, the blue can be extracted from the split RGB (i.e., red, green, and blue) channel images to correct the blank background, resulting in more readable information for the viewer or scanner (Figure 5C and S14A, Supporting Information).

**Figure 6:**
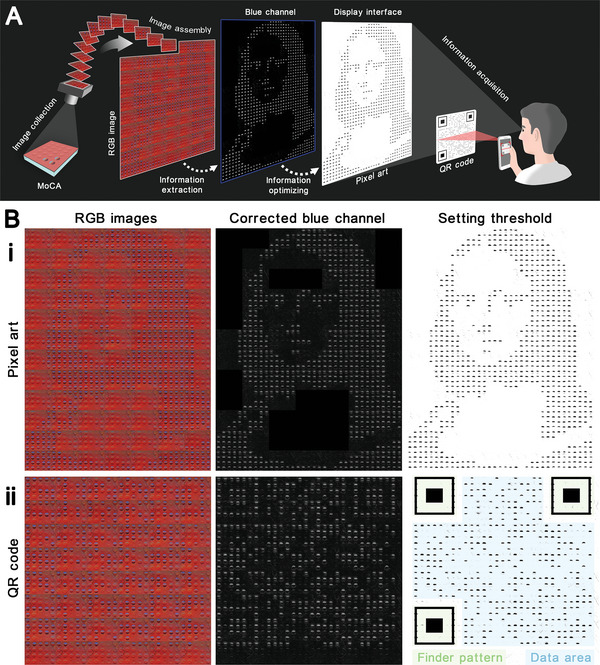
The pixel art drawing and digital coding via the dynamic display of MoCA. A) Schematic illustration shows the MoCA process from image to information. B) Demonstration of (i) anti‐counterfeiting pixel art and (ii) QR code assembled by images from MoCA's dynamic display (light green and light blue backgrounds denote the finder patterns and the data area of the QR code, respectively).

For a static pixelated display, a high resolution is required, in principle, for high information capacity. The more pixels packed into a confined space, the higher resolution the system will have. The resolution of the MoCA depends greatly on the size of MoC and the space between MoCs that can be fabricated. For example, microfabrication, such as soft lithography technology, can minimize the sizes of MoC to hundreds of microns. The MoC array can thus be arranged more closely, which in turn will reduce the limiting resolution to hundreds of microns. By comparison, with a constant resolution, the dynamic pixelated display system enables the continuous output of a series of different patterns. These patterns can be then combined in a specific sequence to compose a more sophisticated pattern containing richer information (**Figure** **6**A). In this way, the information content of an area‐constrained color‐changing system would not be limited by the resolution. As a demonstration, we compose pixel art through the MoCA system (Figure 6Bi and S14B, Supporting Information). This pixel art is composed of 48 MoCA images, containing a total of 1680 controllable pixels (35 × 48 pixels). Among them, 751 specific pixels are tuned to blue to form the pixel painting of the Mona Lisa portrait. The pixels can be clearly rendered without interference between adjacent pixels, demonstrating that high‐resolution pixel patterns can be drawn by MoCA's dynamic display. This dynamically displaying pixel patterns through MoCA can be applied as a distinctive anti‐counterfeiting technique, where the information owners need to secure the label with covert protections unbeknownst to counterfeiters. Herein, the ethanol‐triggered morphing and angle‐dependent coloration can secure the anti‐counterfeit label with the first protective mechanism. The second mechanism comes from the fact that the photopic sensitivity of human vision to red light (*λ* ≈ 620 nm) are ten times higher than that to blue light (*λ* ≈ 450 nm), so it is difficult to accurately identify the constantly appearing blue pixel patterns in MoCA dominated by a red background, especially for patterns with very rich pixels.^[^ ^15^ ^]^ Nevertheless, the covert information in the unperceivable blue pixels can be readily extracted via a blue channel and then clearly presented to the appraiser after an appropriate threshold setting (Figure 6A, Figure 6Bi, and S14B, Supporting Information). Therefore, the anti‐counterfeiting achieved by MoCA can elude the would‐be counterfeiters, but can be authenticated by a custom‐designed scanner in the appropriate environment. When the designed pattern functions as a digital key, such as a quick response code (QR code), the process of securing an anti‐counterfeit label is in fact an encryption process. In a similar approach, we combine MoCA's dynamic display with binary coding to create a QR code (Figure 6Bii). The demonstrated QR code is composed of an array of 29 × 29 pixels. Except for the regular finder patterns that need to be redrawn to enhance the success rate in locating the QR code, the 647 pixels in the data area storing the primary data can be read accurately and efficiently. The data stored in this QR code is a web address that can also be deciphered by the custom‐designed scanner with the appropriate filter. Thus, our MoCA's capability in displaying the human‐eye unperceivable but machine‐readable pixel art and QR code suggests that MoCA's dynamic pixel display could be applied in anti‐counterfeiting and encryption technologies in the future.

## 3 Conclusion

Inspired by the dual‐color micro‐concavity on butterfly wings, we have pixelated the solvent‐responsive structural colors based on a morphable concavity array that combines the angle‐dependent color‐changing 2D‐PC and transformable elastomer actuator. We study the surface topography of the morphable concavity and its corresponding diffraction wavelength to explain the color‐changing mechanism. Based on these findings, we compose the color‐changing concavity into an array and demonstrate its dynamic display applications by editing the colors of the pixels. We believe this pixelating strategy via manipulation of surface topography can further design hierarchical interfaces and multiple optical systems for biomimetics and soft robotics.

## 4 Experimental Section

### Chemicals

Sylgard 184 silicone elastomer kits were purchased from Dow Corning. Graphene nanoplates were purchased from Suzhou TANFENG graphene Tech Co. Ltd. PS‐NPs (diameter ≈ 600 nm) were purchased from Shanghai Huge Biotechnology Co. Ltd. NIPAM, N,N’‐methylene‐bis‐acrylamide (BIS), and trichloro(1H,1H,2H,2H‐perfluorooctyl)silane were purchased from Sigma‐Aldrich. BP, sodium dodecyl sulfate (SDS), and ethanol were purchased from Aladdin Industrial Corporation. Chloroform was purchased from Fisher Scientific. Deionized (DI) water was produced by a DI water system (Milli‐Q, 18.3 MΩ). All chemicals were used as received without further purification. PDMS was prepared by mixing Sylgard 184 silicone elastomer base with 10 wt.% of its curing agent. NIPAM solution was prepared by dissolving 9 wt.% of NIPAM and 0.2 wt.% of BIS in DI water. BP solution was prepared by dissolving 40 wt.% of BP in chloroform. GPDMS was prepared by mixing 7 wt.% BP solution and 2 wt.% graphene nanoplates in PDMS prepolymer.

### Preparation of PC Elastomer Film

GPDMS was spin‐coated on a glass substrate at 300 rpm for 1 min. Then the GPDMS‐coated substrate was cured at 80°C on a heating plate for 15 min. The cured GPDMS film (thickness ≈ 250 µm) was placed in a clean Petri dish and immersed in deionized water after 5 min plasma treatment. To fabricate the PC elastomer, the PS‐NPs suspension (PS‐NPs : deionized water : ethanol = 1:1:1 in the volume ratio) was gently dripped onto the air‐water interface until a uniform monolayer of PS‐NPs assembly film was formed on the water surface. Subsequently, 10 µL SDS solution was dripped along the edge of the Petri dish to closely pack the monolayer of PS‐NPs through a self‐assembly process. By sucking out excess water carefully, the two‐dimensional photonic crystal from the close‐packed PS‐NPs colloidal array was transferred to the GPDMS. Eventually, the PC elastomer film was obtained after evaporating the residual water at room temperature for 2 h and then annealing in an oven at 65 °C for 30 min.

### Preparation of MoCA

PC elastomer film was flipped over and placed on a clean PDMS substrate to protect the PC‐assembled side. A PDMS partition with a circular hole array (5 × 5 array, diameter of each hole = 3 mm, space between holes = 1.5 mm) was fabricated by punching a bulk PDMS (27 mm × 27 mm × 3.5 mm) with a puncher (diameter = 3 mm). The PDMS hole array was then bonded on the blank side of the PC elastomer film via wet PDMS naturally curing at room temperature to form a well array. To modify the hydrogel layer at the bottom of PDMS wells, NIPAM solution was filled into the wells and placed in a UV chamber (Wavelength = 365 nm, Power = 12.8 mW (cm^2^)^−1^, ABM‐USA, Inc.) for 30 min irradiation. The UV light excited the doped BP in GPDMS to initiate the graft polymerization of NIPAM. Thereafter, a layer of pNIPAM was grafted onto the GPDMS blank surface at the bottom of each well. The residual NIPAM solution was removed, and the wells were rinsed with DI water three times. Eventually, the whole assembly was detached from the PDMS substrate to obtain the MoCA device.

### Microscopy

The PC‐EA film at the concavity area was imaged using a scanning electron microscope (Hitachi S4800N, Japan). Energy‐dispersive X‐ray scattering was used to obtain the elemental mapping of various elements in the cross‐section of the trilayer.

### Topography Test of MoC

The MoCA device was placed on the testing stage of a profiler (DektakXT Stylus Profiler, Bruker, USA). 100 µL of ethanol solutions were injected into the hole underneath the specific MoC at a flow rate of ≈600 µL min^−1^ using tubing. After ethanol triggered and formed the concavity, the ethanol solution was removed. The environment was then rapidly warmed to about 60 °C by a heating plate. As the ethanol evaporated, the MoC's surface gradually returned to flatness. The profile of the concavity and depth at the center of concavity during the MoC's transformation induced by ethanol treatment and heating was obtained by the profiler. The reproducibility test was repeated for 10 cycles alternating between ethanol and heat treatments.

### Finite Element Analysis for the MoC Morphing

A single MoC was studied via simulation. The geometry model of the MoC was constructed according to the designed dimensions. To reduce the computation cost and error, only 1/2 of the MoC geometry was simulated. The GPDMS and the pNIPAM were assumed linear elastic materials due to the limited deformation in this case. The pNIPAM layer was assumed to undergo isotropic and homogeneous expansion upon swelling. The displacement and stress on the MoC at various expansion amplitudes were studied.

### Color Switching and Diffraction Spectrum Testing of PC Elastomer Film and MoC

The camera (EOS 70D, Canon) and white light LED (23 cm × 16 cm, 100 W) were set on the same side of the normal line to the PC elastomer plane. The incident angle of the light was ≈60°, while the observation angle of the camera was about 20°. Scattering spectra of the PC elastomer film and MoC were measured by an angle‐resolved spectrum system (R1, Ideaoptics, China).

### Dynamic Display of the MoCA

The same number of tubings as the MoC array were connected to the bottom of each MoC. Each microtubing was used to control the deformation of one MoC. When writing on MoCA, 100 µL of ethanol was introduced to the specific MoC at a flow rate of 600 µL min^−1^ to trigger its concave state. The order of injecting ethanol into MoC followed the order in which the letter or the pattern was written or drawn. After all designated MoCs had morphed and changed color, the bottom of the MoCA device was treated with hot air at ≈60 °C to evaporate the ethanol so as to erase the written letter or drawn pattern. The same writing and erasing processes were repeated when the next letter or pattern was written or drawn.

## Conflict of Interest

H.C. Shum is a scientific advisor of EN Technology Limited, and MicroDiagnostics Limited, in both of which he owns some equity, and also a managing director of the research centre, namely Advanced Biomedical Instrumentation Centre Limited. The works in the paper is however not directly related to the works of these two entities, as far as we know.

## Supporting information

Supporting Information

Supplemental Movie 1

Supplemental Movie 2

Supplemental Movie 3

Supplemental Movie 4

Supplemental Movie 5

Supplemental Movie 6

## Data Availability

The data that support the findings of this study are available in the supplementary material of this article.
