# Supplementary material for: Pixelating Responsive Structural Color via a Bioinspired Morphable Concavity Array (MoCA) Composed of 2D Photonic Crystal Elastomer Actuators
Source: Adv Sci (Weinh). 2023 Feb 15;10(11):2300347. doi: 10.1002/advs.202300347 (PMC10104634; doi:10.1002/advs.202300347)
Supplement: Supplementary file 1 — Supporting Information [file ADVS-10-2300347-s004.pdf]

Supporting Information

Pixelating responsive structural color via a bioinspired morphable concavity array composed of 2D photonic crystal elastomer actuators

Yi Pan, Chang Li, Xiaoyu Hou, Zhenyu Yang, Mingzhu Li, and Ho Cheung Shum**

1. Supplementary notes

Note S1:

The 2D diffraction condition:[14e]

$$d\frac{\sqrt{3}}{2}(sin\theta_{out} + \sin\theta_{in}) = n\lambda \qquad (1)$$

where d is the spacing between nearest neighboring particles, θ_{in} and θ_{out} are the incidence and diffraction angles from the normal, n is the diffraction order, and λ denotes the wavelength of diffraction light.

In our case, d = 600 nm and n = 1, so (1) can be expressed as:

$$600 \times \frac{\sqrt{3}}{2}(sin\theta_{out} + \sin\theta_{in}) = \lambda \qquad (2)$$

Since the wavelength range we are discussing is limited to visible light, the values of λ are between 450-750 nm, i.e., $\lambda \in [450 ,750]$. In addition, both θ_{in} and θ_{out} are on the same side of the normal to the 2D-PC plane, so they range from 0° to 90°, i.e., $\theta_{in} \in [0 ,90]$ and $\theta_{out} \in [0 ,90]$. Therefore, we can specify λ to take the values of 450, 500, 550, 600, 650, 700, and 750 nm. At a specific value of λ, the relationship between θ_{in} and θ_{out} can be derived based on (2) and plotted on the θ_{in}-θ_{out} diagram (Figure 4B).

Note S2:

Based on the definition of angular parameters (θ_{in}, θ_{out}, and $\theta_{incl.}$) and the geometric relationship between them, the angles of incidence and diffraction after a certain inclination can be calculated as:

$$\theta'_{in} = \theta_{in} - \theta_{incl.}$$

$$\theta'_{out} = \theta_{out} - \theta_{incl.} \qquad (3)$$

where θ_{in} and θ_{out} are the initial angles of incidence and diffraction of a certain subdivided plane on MoC, θ'_{in} and θ'_{out} are the corresponding angles of the plane after inclination, and $\theta_{incl.}$ denotes the angle of inclination.

Under the condition that the initial angles are $\theta_{in} = 60°$ and $\theta_{out} = 20°$, we can substitute them into (3) to obtain:

$$\theta'_{in} = 60° - \theta_{incl.}$$

$$\theta'_{out} = 20° - \theta_{incl.} \qquad (4)$$

Therefore, from (4), it can be deduced that

$$\theta'_{out} = \theta'_{in} - 40° \qquad (5)$$

Furthermore, from the test results of Figure 3C, we know the possible existence of $\theta_{incl.}$ in MoC ranges from -20° to 20°. Combining $\theta_{incl.} \in [-20{,}20]$ with (5), we can further conclude that the angular parameters after inclination satisfy (5) and take values in the ranges as follows:

$$\theta'_{in} \in [80\ ,40\ ] \quad and \quad \theta'_{in} \in [40\ ,0\ ]$$

Based on the above calculations, we can trace the function curve of (5) in the θ_{in}-θ_{out} diagram, as shown in Figure S8.

Extracting the intersection of the function curve of (5) and the calculated wavelength curves, we can get the θ'_{in} and θ'_{out} corresponding to each intersection point and then deduce the corresponding $\theta_{incl.}$ from (4). Thus, the relationship between the diffraction wavelength and $\theta_{incl.}$ can be found and plotted in Figure 4H.

2. Supplementary figures

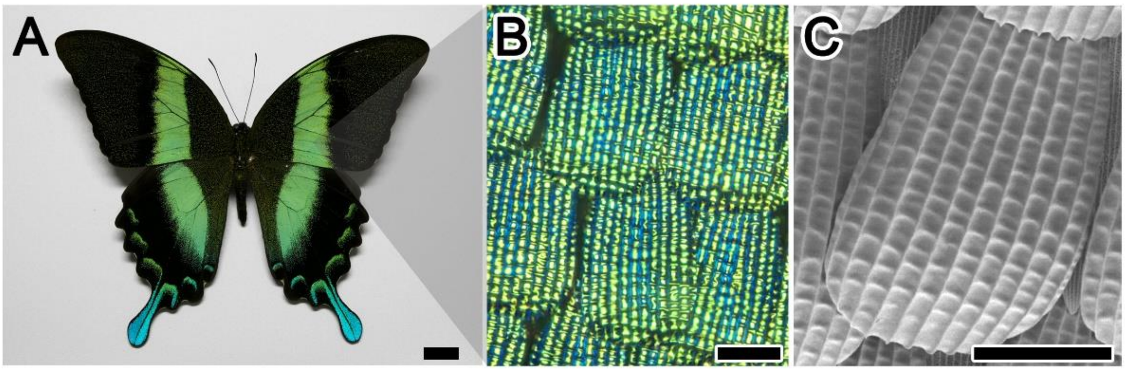

Figure S1. Images of the *Papilio palinurus* butterfly. (A) Photograph of a *Papilio palinurus* butterfly. Scale bar: 1 cm. (B) Real-color image of the butterfly wing scales. Scale bar: 50 μm. (C) Electron scanning microscope (SEM) image of single pieces of wing scale. Scale bar: 50 μm.

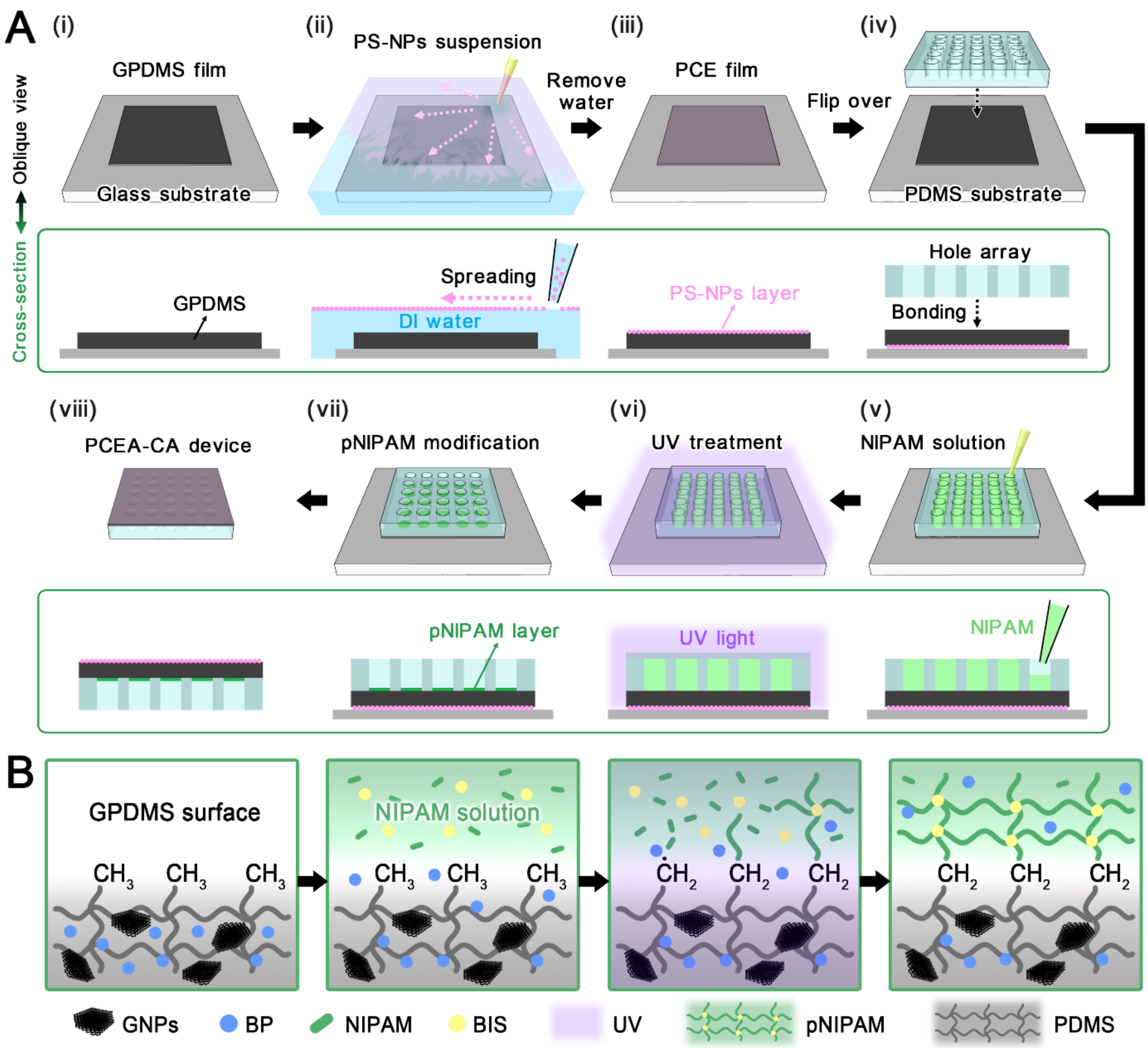

Figure S2. Schematic illustrations of (A) MoCA preparation (Oblique view and the corresponding cross-section) and (B) the process of pNIPAM grafting onto GPDMS surface, i.e., the process of step (v) to (vii) in (A). GNPs: graphene nanoplates; BP: benzophenone; NIPAM: N-isopropylacrylamide; UV: ultraviolet light; pNIPAM: poly(N-isopropylacrylamide); PDMS: polydimethylsiloxane.

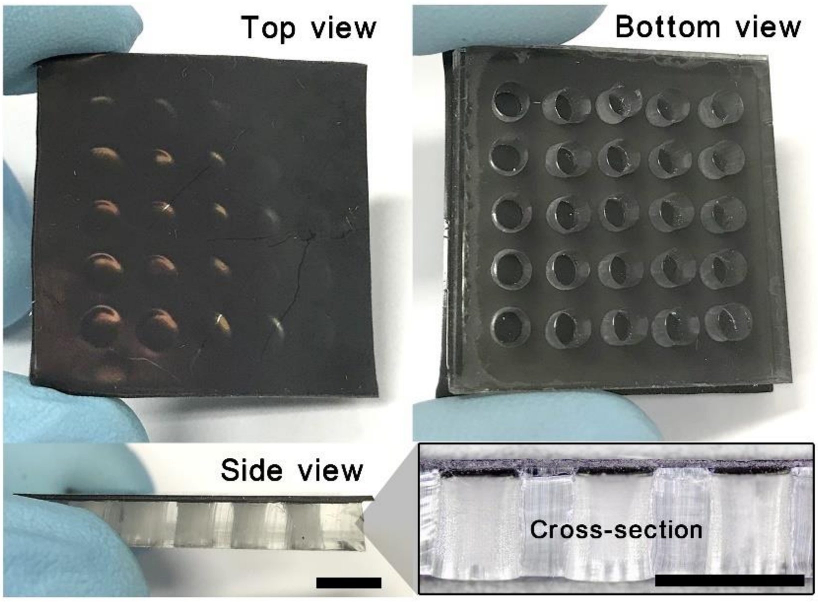

Figure S3. Photographs of MoCA. Scale bars: 5 mm.

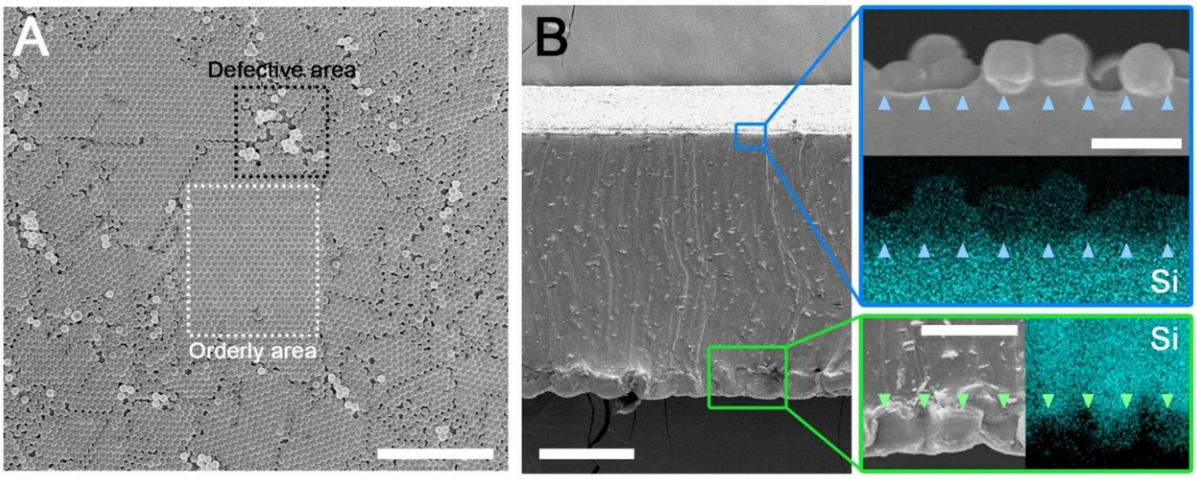

Figure S4. SEM images and EDX mapping of PC-EA film. (A) SEM image of the top view of the PS-NPs layer. Scale bar: 10 μm. (B) The cross-section SEM image of PC-EA film at the concavity area and the EDX mapping for the top and bottom regions (interfaces are marked with arrows). Scale bar of PC-EA film: 100 μm. Scale bar of PS-NPs layer (top region): 1 μm. Scale bar of pNIPAM layer (bottom region): 50 μm.

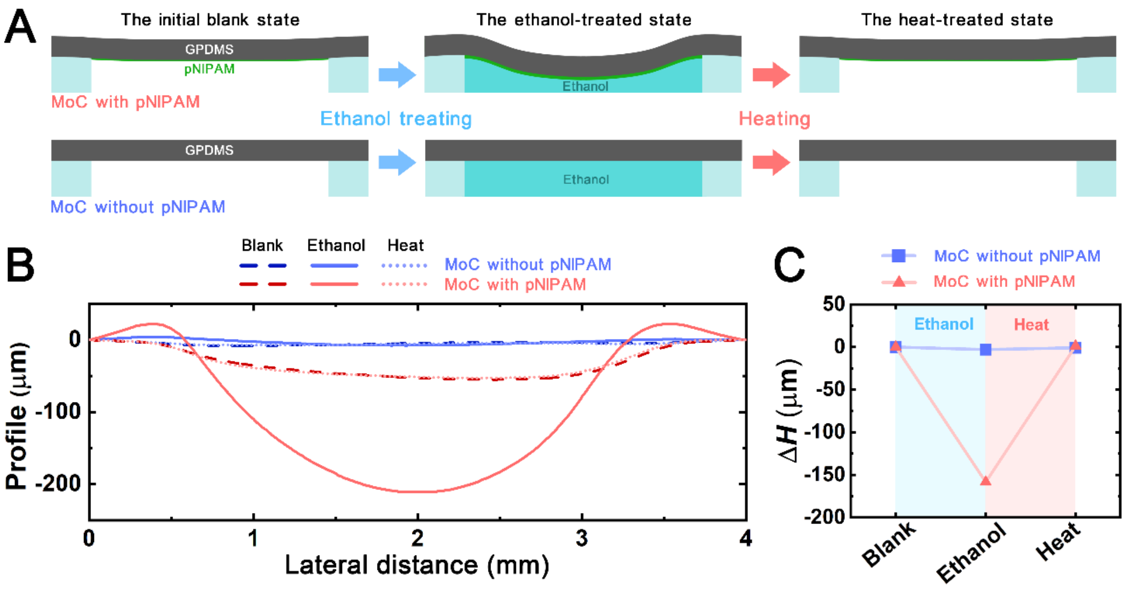

Figure S5. Comparison of the deformation of MoCs with and without pNIPAM layers upon ethanol treating and heating stimulations. (A) Schematic illustration of the MoCs upon stimulations. (B) Profiles of the MoCs' concave areas in their blank, ethanol-treated, and heating states. (C) Δ*H* change of the MoCs in their blank, ethanol-treated, and heating states.

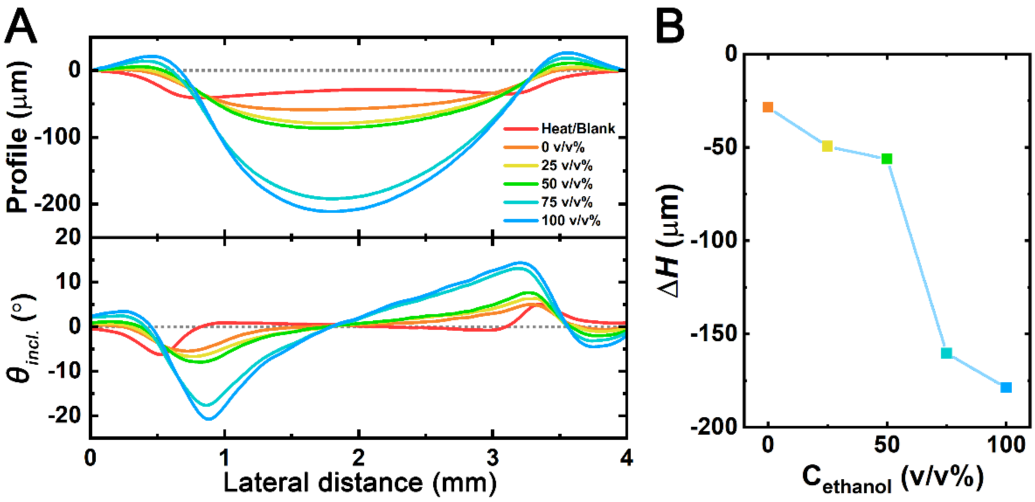

Figure S6. Comparison of MoC deformation at different ethanol concentrations. (A) Profiles and the corresponding inclination angles of the concave area under the stimulation of different concentrations of ethanol solutions. (B) Relationship between the ΔH of MoC and the corresponding ethanol concentrations used in the treatments.

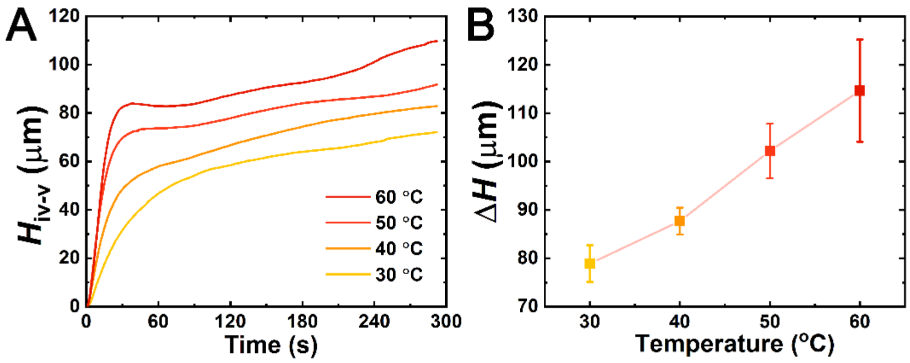

Figure S7. Comparison of MoC deformation at different temperature treatments. (A) The rise of the MoC center point height (*H*) from the inflection point for 5 min (i.e., from stage iv to v) at different ambient temperatures. (B) The rising height of MoC (Δ*H*) from its lowest value for 5 min (i.e., from stage iii to v) at different ambient temperatures.

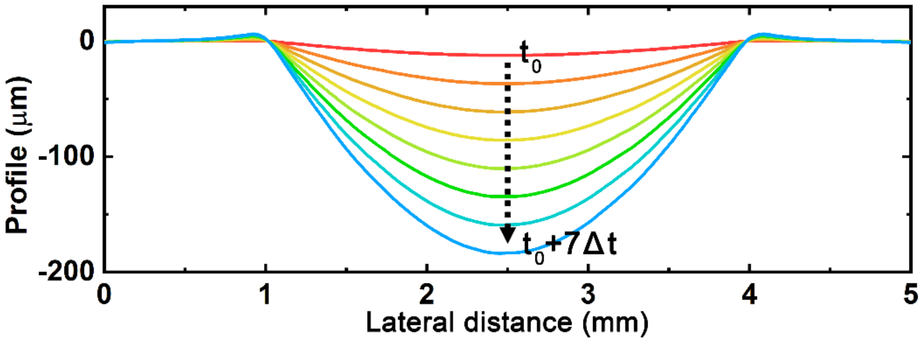

Figure S8. Finite element analysis simulates the profile of the MoC as a function of time.

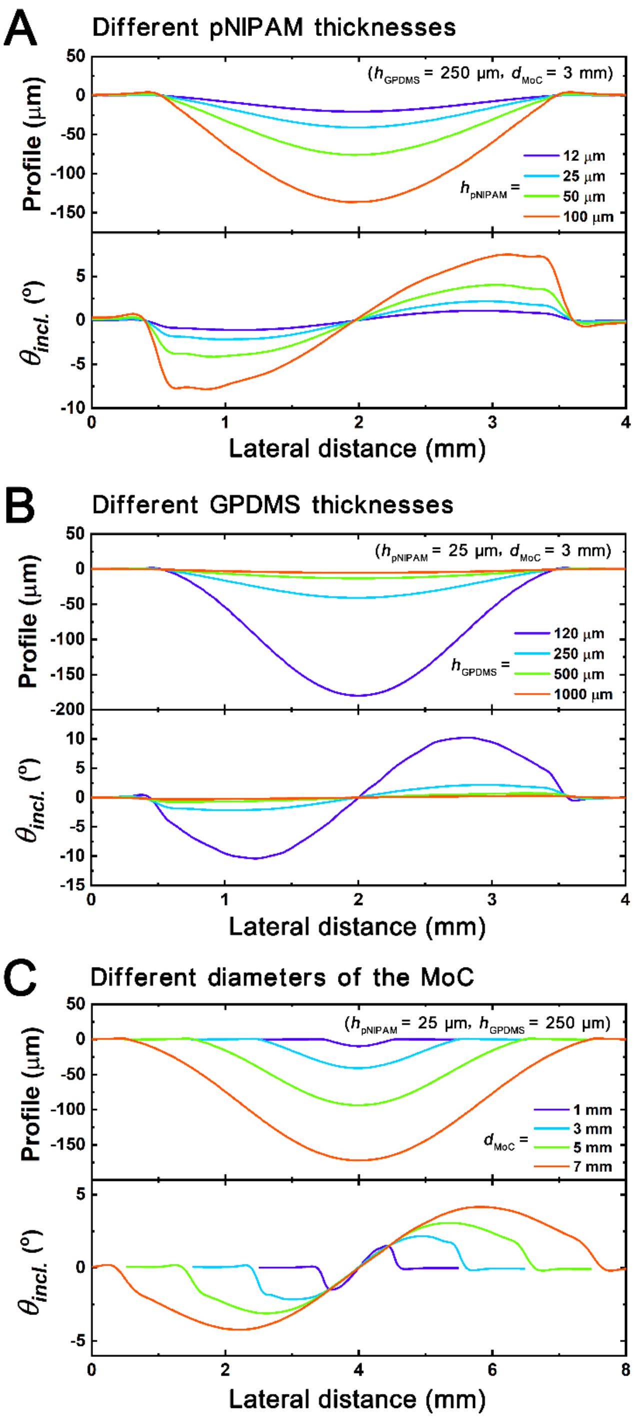

Figure S9. Simulated deformation profiles and $\theta_{incl.}$ of MoC at (A) different pNIPAM layer thicknesses, (B) GPDMS layer thicknesses, and (C) MoC diameters under the same actuating conditions. h_{GPDMS}, h_{pNIPAM}, and d_{MoC} are defined as the thickness of the GPDMS layer, the thickness of the pNIPAM layer, and the diameter of MoC, respectively.

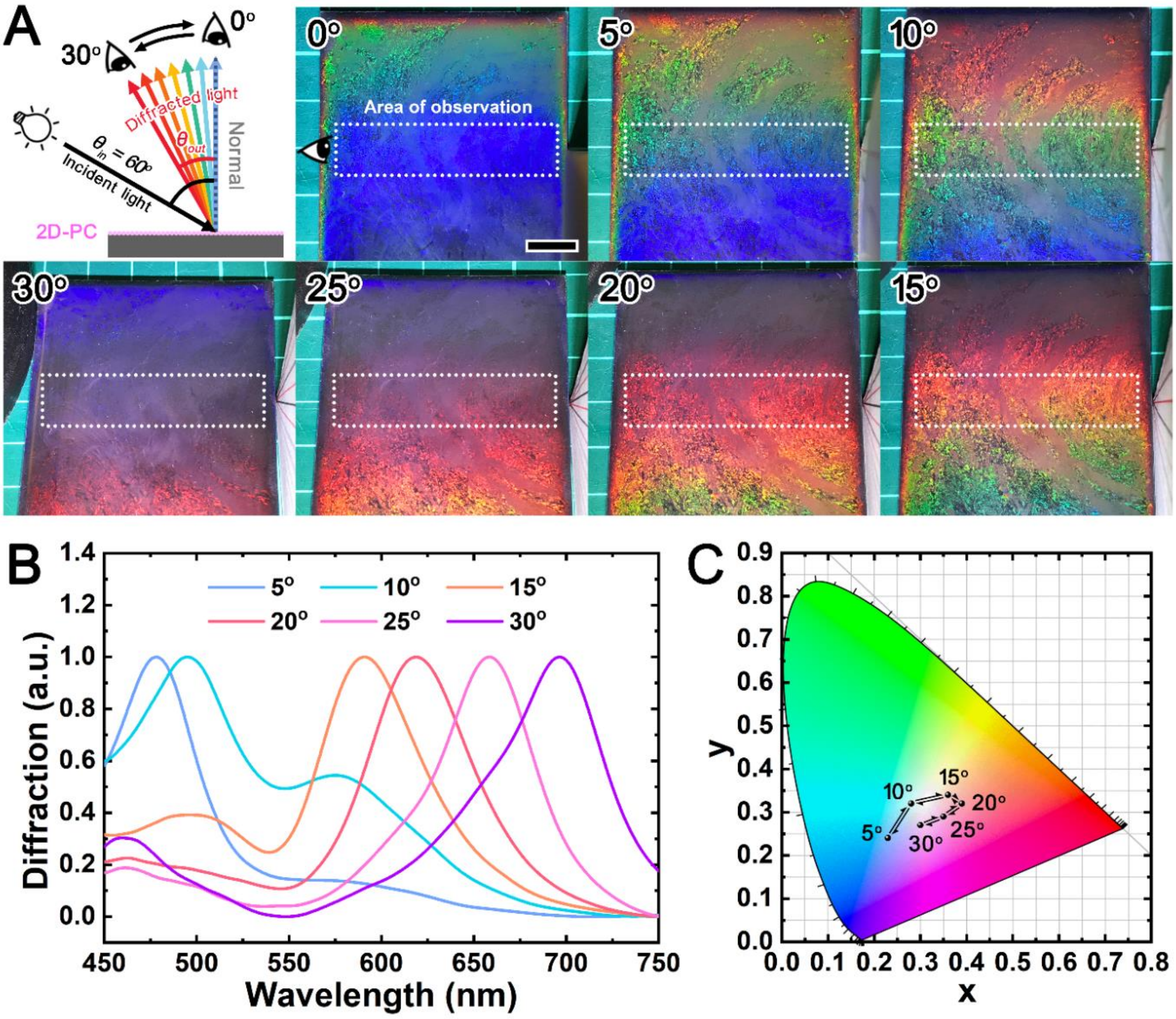

Figure S10. Angle-dependent color-changing 2D-PC. (A) Schematic illustration and photographs shows the color shifts with various diffraction or observation angles (θ_{out} = 0° ~ 30°) at a fixed incident angle (θ_{in} = 60°) within the plane containing the incident and diffracted lights for 2D-PC. Scale bar: 1 cm. (B) The spectra of the 2D-PC from different diffraction angles (θ_{out} = 5°~30°) at a fixed incident angle of 60°. (C) The CIE chromaticity diagram of (A).

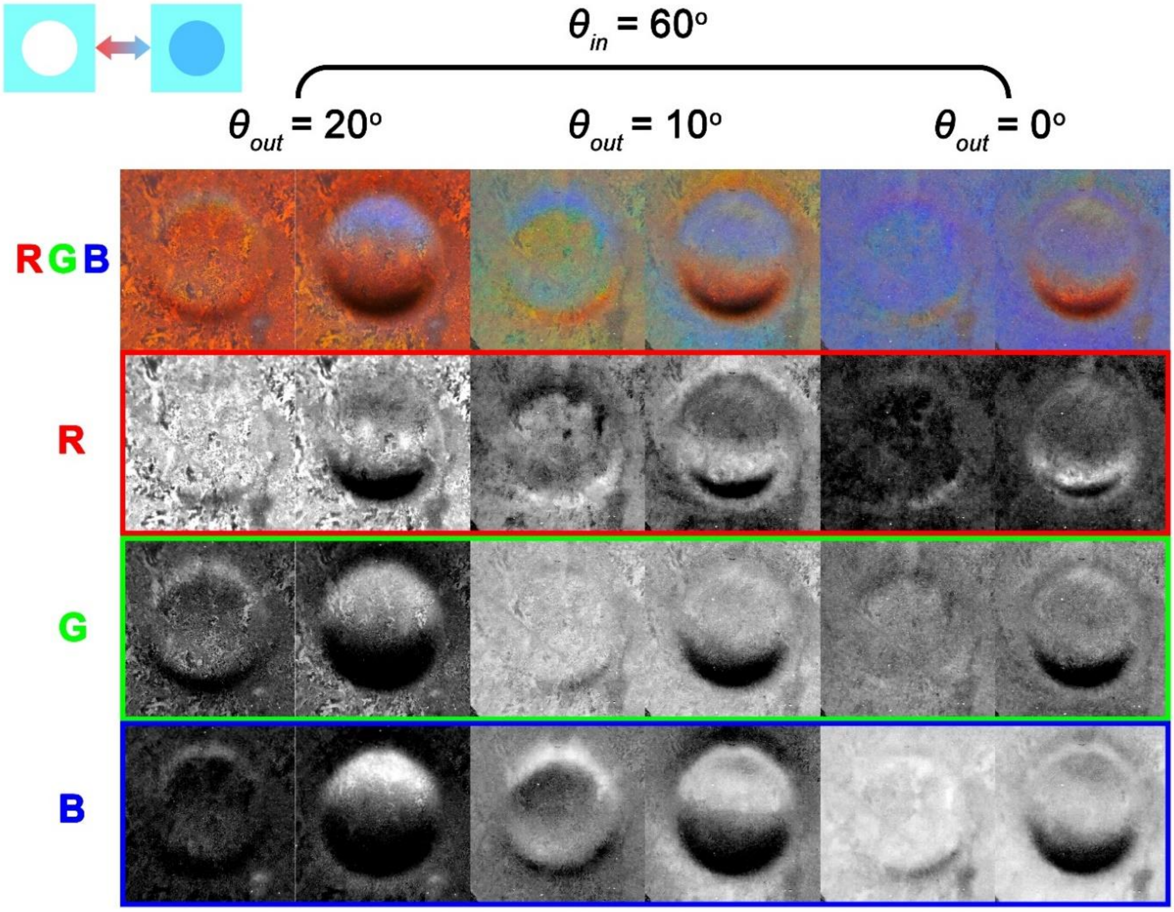

Figure S11. Color switching of MoC from different diffraction angles (θ_{out} = 0°, 10°, and 20°) at a fixed incident angle (θ_{in} = 60°) and their corresponding red (R), green (G), and blue (B) channel images.

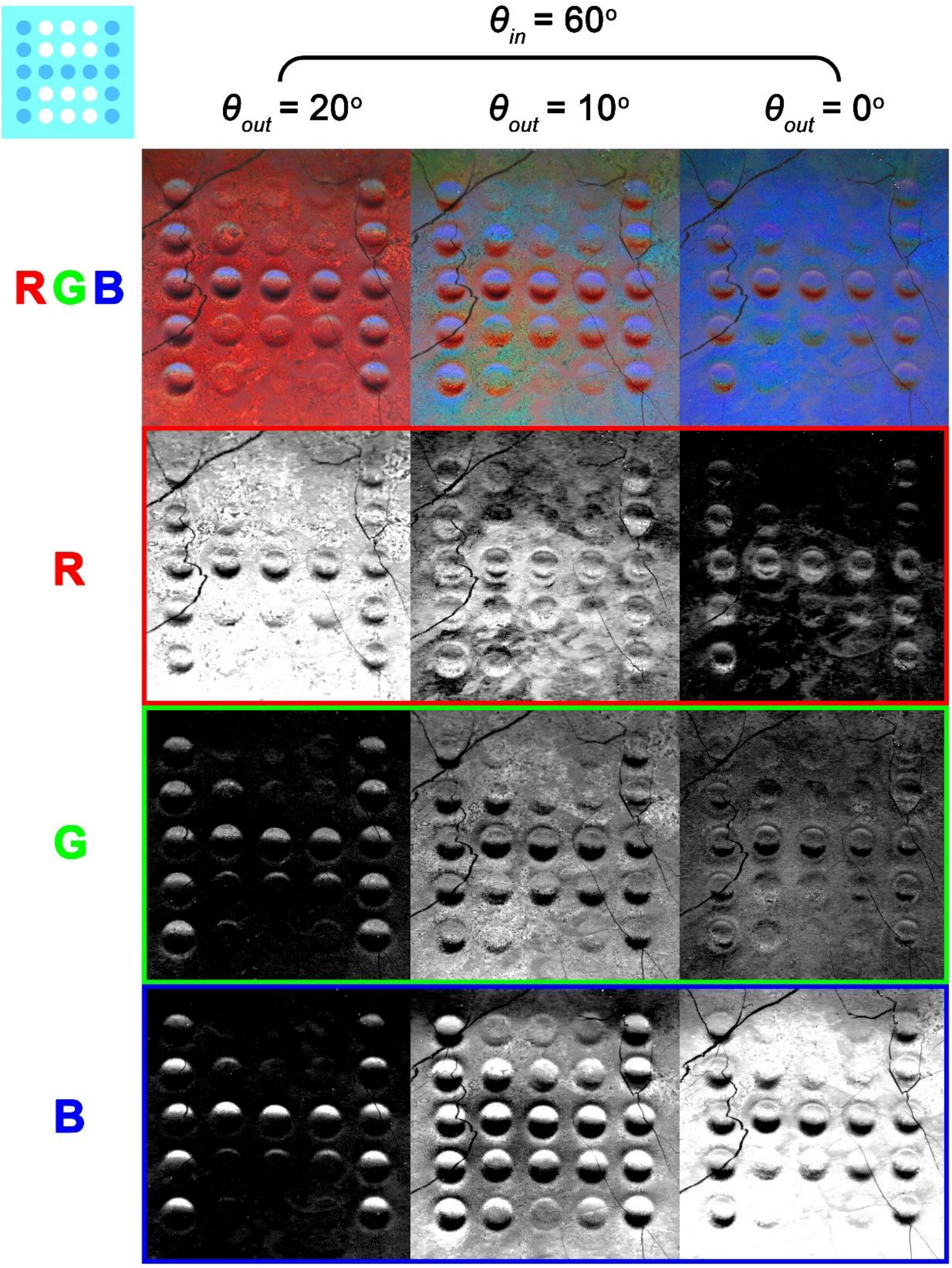

Figure S12. MoCA's RGB images showing letter "H" from different diffraction angles (θ_{out} = 0°, 10°, and 20°) at a fixed incident angle (θ_{in} = 60°) and their corresponding red (R), green (G), and blue (B) channel images.

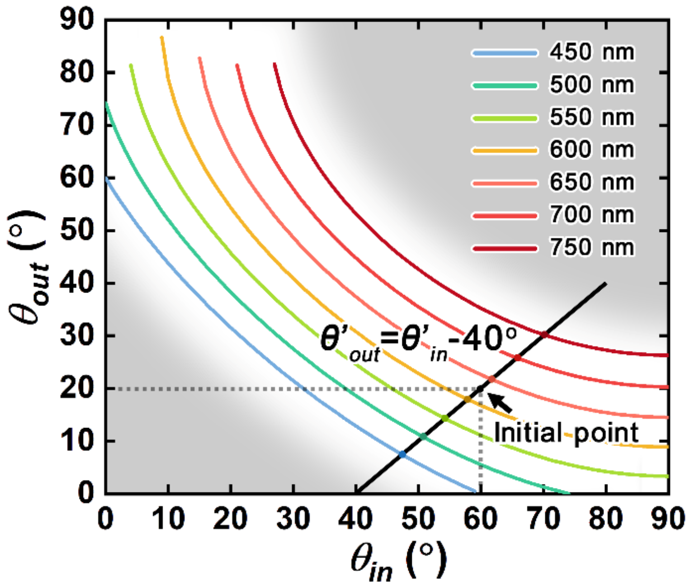

Figure S13. The theoretical diffraction wavelength variation of the subdivided plane on MoC (θ_{in} = 60°, θ_{out} = 20°, and $\theta_{incl.} \in [-20,20]$) is located on the graph (black line) of the linear equation $\theta'_{out} = \theta'_{in} - 40°$ in the θ_{in}-θ_{out} diagram. (The gray background indicates the non-visible area.)

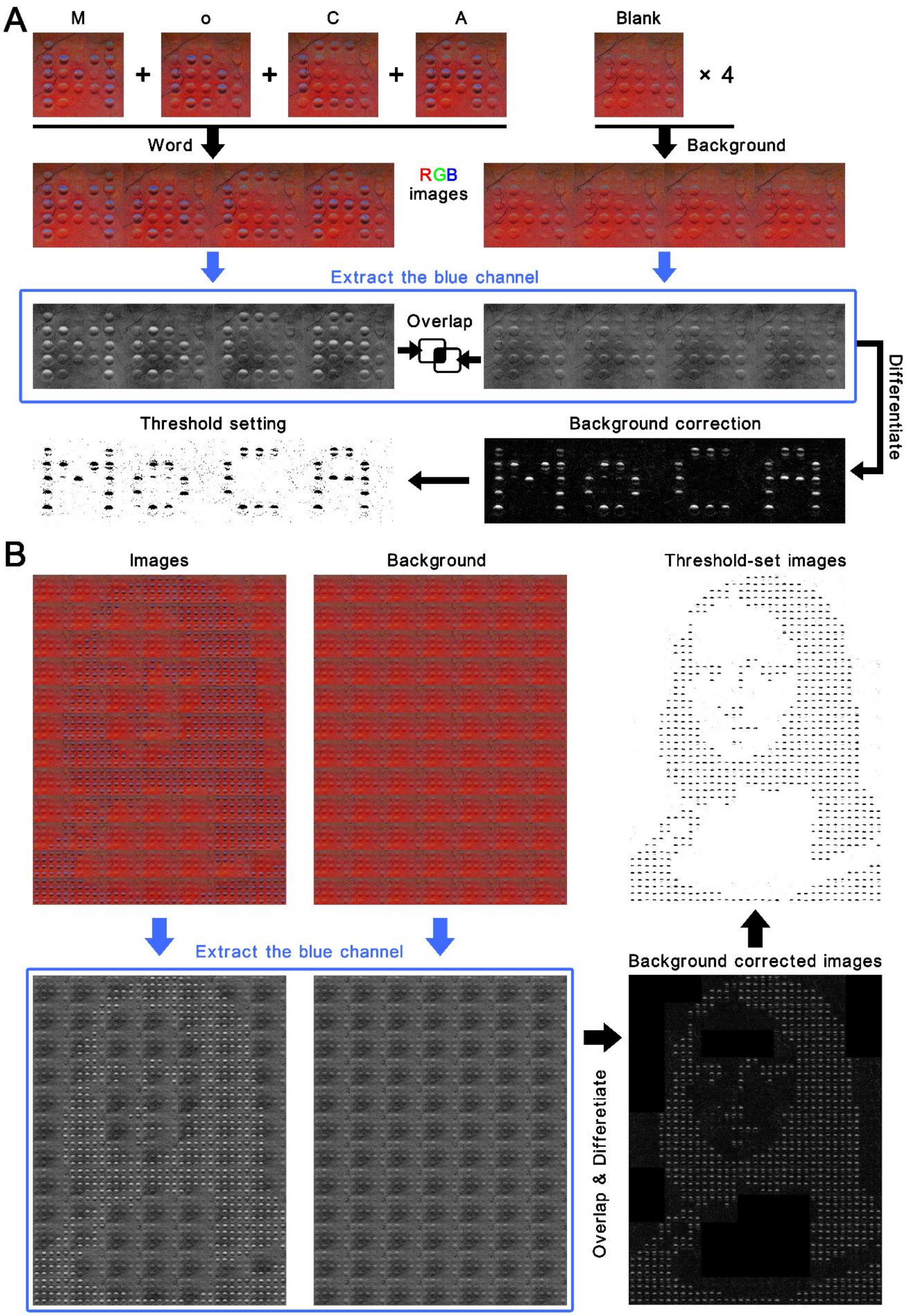

Figure S14. Background correction processes for (A) word and (B) pixel art composed of images dynamically displayed by MoCA.

3. Supplementary tables

Table S1. The swelling rate of PDMS and pNIPAM towards anhydrous ethanol.

Materials	Swelling rate[a)]	Ref.
PDMS	1.04	[13c]
	~ 1.1	[13e]
	~ 1.1	[13d]
pNIPAM	~ 4.0	[13b]
	~ 4.0	[13a]
	~ 2.5	[13f]

[a)] The swelling rate is measured by X/X_0, where X is the geometric parameter (i.e., length, height, or volume) of the testing sample (i.e., PDMS or pNIPAM) in the anhydrous ethanol and X_0 is the same geometric parameter of the dry testing sample.

4. Supplementary movies

Movie S1. Stimuli-responsive topographical change of the MoC.

Movie S2. Stress distribution of the MoC by finite element analysis.

Movie S3. Angle-dependent color change of the PC elastomer film.

Movie S4. Color switching of the MoC and the change of its central height.

Movie S5. Cyclic test of MoC’s color switching.

Movie S6. Dynamic display of MoCA.
